# Novel Alzheimer's disease subtypes based on functional brain connectivity in human connectome project

**DOI:** 10.1038/s41598-024-65846-z

**Published:** 2024-06-27

**Authors:** Jinhua Sheng, Yu Xin, Qiao Zhang, Ze Yang, Luyun Wang, Qian Zhang, Binbing Wang

**Affiliations:** 1 School of Computer Science and Technology, Hangzhou Dianzi University, Hangzhou, 310018 China; 2 Key Laboratory of Intelligent Image Analysis for Sensory and Cognitive Health, Ministry of Industry and Information Technology of China, Hangzhou, 310018 China; 3 Beijing Hospital, Beijing, 100730 China; 4 National Center of Gerontology, Beijing, 100730 China; 5 Institute of Geriatric Medicine, Chinese Academy of Medical Sciences, Beijing, 100730 China

**Keywords:** Alzheimer’s disease, Functional connectivity, HCP MMP, Individual differences, Limbic system, Resting-state fMRI, Neuroscience, Alzheimer's disease

## Abstract

The pathogenesis of Alzheimer's disease (AD) remains unclear, but revealing individual differences in functional connectivity (FC) may provide insights and improve diagnostic precision. A hierarchical clustering-based autoencoder with functional connectivity was proposed to categorize 82 AD patients from the Alzheimer's Disease Neuroimaging Initiative. Compared to directly performing clustering, using an autoencoder to reduce the dimensionality of the matrix can effectively eliminate noise and redundant information in the data, extract key features, and optimize clustering performance. Subsequently, subtype differences in clinical and graph theoretical metrics were assessed. Results indicate a significant inter-subject heterogeneity in the degree of FC disruption among AD patients. We have identified two neurophysiological subtypes: subtype I exhibits widespread functional impairment across the entire brain, while subtype II shows mild impairment in the Limbic System region. What is worth noting is that we also observed significant differences between subtypes in terms of neurocognitive assessment scores associations with network functionality, and graph theory metrics. Our method can accurately identify different functional disruptions in subtypes of AD, facilitating personalized treatment and early diagnosis, ultimately improving patient outcomes.

## Introduction

Alzheimer's disease (AD) is a complex neurodegenerative disorder that affects up to 50 million patients^1^, with projections indicating an increase to 131.5 million by the year 2050^2^. It’s characterized by progressive cognitive decline, memory loss, and behavioral changes. The degenerative process associated with this disease can extend over several years, presenting a substantial burden on individuals, society, and the economy at large. It’s noteworthy that AD lacks a uniform standard but comprises several subtypes, each with unique clinical and neuropathological features. Some individuals may exhibit pronounced memory loss, while others may manifest more noticeable emotional issues or executive function impairments. Furthermore, the diversity in terms of brain pathology and responses to treatment complicates the early diagnosis and management of AD. Therefore, it is imperative to deepen our understanding of the heterogeneity of this disease, viewing it as a personalized issue, to enhance diagnostic accuracy, prognosis, and treatment strategies.

The development of neuroimaging techniques has enabled researchers to further investigate brain changes in AD patients. Among these, structural magnetic resonance imaging (sMRI) can be used to assess brain volume, regional characteristics, cortical thickness, and curvature^3^. Functional magnetic resonance imaging (fMRI) enables the detection of dynamic changes in brain activity and metabolism^4^. Additionally, Positron Emission Tomography (PET) unveils molecular metabolism, aiding in the early detection of lesions and abnormalities^5^. Researchers have employed neuroimaging data to explore the intricacies of these AD subtypes. For example, Hwang^6^ utilized Ward clustering on structural MRI data from AD patients, resulting in the identification of three subtypes of cortical thinning: medial temporal, diffuse, and parietal-dominant. Young^7^ used a combination of clustering and event-based modeling to define three spatiotemporal atrophy patterns in AD: originating from the medial temporal lobe, temporofrontal regions, and the basal ganglia. In a study conducted by Whitwell^8^, a cluster analysis is performed on 62 amyloid-positive AD dementia patients to investigate the changing pattern of AD in the entorhinal cortex and neocortex.

**Figure 1:**
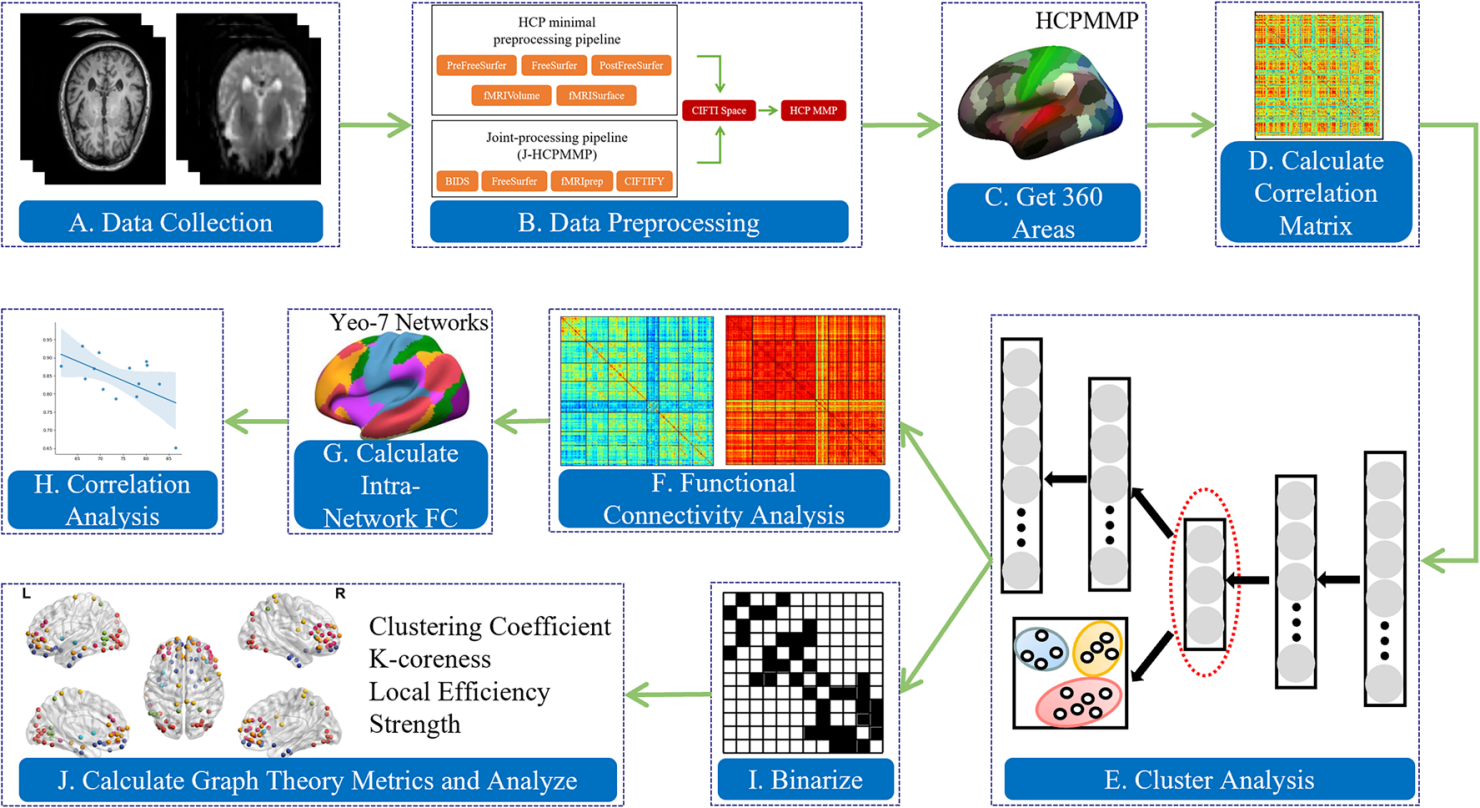
Research methods flowchart.

Existing research on AD subtypes is largely based on sMRI or PET imaging. In this study, we investigate individual functional connectivity (FC) differences in AD patients using resting-state fMRI. Aiming for a precise understanding of subtype differences, we applied the Human Connectome Project Multi-Modal Parcellation (HCP MMP) to ADNI data to provide a more detailed brain region segmentation. Subsequently, we calculated Pearson correlation coefficients of time series to construct a correlation matrix and feed the upper triangle of the matrix into an autoencoder for learning. Hierarchical clustering was performed on AD patients in the low-dimensional latent space obtained from autoencoder learning. Finally, potential differences among AD subtypes were investigated using methods such as Generalized Linear Models (GLM)^9,10^ and graph theory. The study has identified two distinct subtypes exhibiting considerable heterogeneity: one showed significant brain-wide decrease in functional connectivity, and the other marked by significant functional impairment solely within the Limbic System (LS). These findings offer fresh perspectives suggesting that preserving the functionality of the LS could potentially serve as an effective strategy to delay disease progression. Furthermore, our investigation delved into the possible correlations between intra-network FC and clinical cognitive manifestations, yielding intriguing results that furnish valuable insights into the interplay between network dysfunction and clinical presentations. The overall process of the study is shown in Fig. 1.

## Materials and methods

Data collection and sharing for this study is funded by the Alzheimer's Disease Neuroimaging Initiative (ADNI, http://adni.loni.usc.edu). All methods are carried out in accordance with relevant guidelines and regulations. All experimental protocols are approved by the institutional review board (IRB) at Hangzhou Dianzi University (IRB-2020001) and the ethics committee at Beijing Hospital (2022BJYYEC-375-01).

ADNI is managed by various organizations including the National Institute of Biomedical Imaging and Bioengineering (NIBIB), the U.S. Food and Drug Administration (FDA), and the National Institute on Aging (NIA). Its primary objective is to advance knowledge regarding the pathophysiology of AD, develop biomarkers for the disease, enhance diagnostic techniques for early AD detection, and refine clinical trial methodologies.

### Subjects

ADNI is managed by various organizations including the National Institute of Biomedical Imaging and Bioengineering (NIBIB), the U.S. Food and Drug Administration (FDA), and the National Institute on Aging (NIA). Its primary objective is to advance knowledge regarding the pathophysiology of AD, develop biomarkers for the disease, enhance diagnostic techniques for early AD detection, and refine clinical trial methodologies.

A total of 82 AD patients and 50 control normal (CN) individuals, 65 men and 67 women, with ages ranging from 55 to 90, were involved in the study. Each participant underwent both resting-state fMRI and T1 weighted MRI scans. Additionally, the research team gathered demographic information of each participant, such as age, gender, education level. Neurological examination scale scores, including Mini-Mental State Examination (MMSE) and Rey Auditory Verbal Learning Test (RAVLT), were also obtained.

### Data preprocessing

The Human Connectome Project (HCP) proposed a more accurate and detailed method for multimodal brain partitioning, HCP MMP^11^, which divides the left and right brain hemispheres of healthy people into 180 regions. However, The preprocessing pipeline of HCP requires high-quality structural data, functional data, and magnetic field distribution information, etc., which is quite difficult for the ADNI database.

To overcome this hurdle and partition the ADNI dataset into HCP MMP fine brain regions, we employed a preprocessing method called J-HCPMMP (Joint-processing HCP MMP)^12^. This method combines multiple MRI data processing techniques. Different imaging modalities, imaging devices, and parameters can have a significant impact on experiments. To address this, Gorgolewski et al.^13^ from Stanford University proposed using a unified standard to manage and validate complex neuroimaging data. This algorithm converts DICOM files collected from imaging devices into NIFTI format and manages the data based on specific subject visit times and modality types. This facilitates the integration and comparison of multi-modal data from repeated follow-up visits. Therefore, in the J-HCPMMP preprocessing framework, we first implemented the BIDS^13^ and validated the integrity of the ADNI data. In terms of MRI, we categorize DICOM files into structural, functional, and diffusion-weighted imaging, including equipment parameter information used for fMRI artifact detection.

The most popular program for examining structural and functional imaging biomarkers is called FreeSurfer^14^. J-HCPMMP uses Freesurfer to preprocess structural data, including steps such as noise removal and motion correction. Meanwhile, JHCPMMP preprocessed fMRI data using fMRIprep^15^. The specific steps involved using AFNI^16^ for slice-timing correction, interpolating all slices to the midpoint of each echo time; employing FSL^17^ for six-parameter rigid-body motion correction; performing generic distortion correction based on device parameter information in the BIDS dataset; aligning sMRI and fMRI using the boundary-based registration^18^ algorithm; and finally, transforming from the original sampling space to the MNI space using the ANTs^19^. Once the preprocessing was completed, we aligned the structural and functional data to the CIFTI grayscale space, effectively integrating both datasets into a unified coordinate system. Within the CIFTI space, we segmented the cortical apex and subcutaneous tissue. The time series data were obtained by averaging within each specific brain region. This averaging method helps to reduce noise and enhance the reliability of the functional connectivity analysis conducted in our study. By calculating the Pearson correlation coefficients between these brain region sequences, we derived a $360\times 360$ correlation matrix.

### Clustering analysis

A[$\text{N}\times \text{N}]$ matrix is generated by calculating the correlation between each pair of brain regions, where N represents the total number of brain regions. Given the correlation matrix's symmetry, typically only the upper triangular part is utilized as FC data in subsequent studies. This triangular part is converted into a vector format. Hierarchical clustering is a widely employed method for clustering that requires calculating the distance or similarity between each pair of samples. However, as the data dimension increases, distance calculations become more complex and time-consuming. Additionally, in high-dimensional spaces, data tends to become sparse, rendering distance calculations less reliable and leading to less accurate outcomes.

Deep learning has gained significant popularity in brain functional connectivity and network analysis^20,21^. It enables the exploration of connection patterns and network structures among various brain regions, thereby providing valuable insights into brain function and cognitive processes. An unsupervised technique called autoencoder^22^ is commonly used for data dimensionality compression and feature expression. By compressing input data into a lower-dimensional latent space and then reconstructing the original data, autoencoder effectively reduce dimensionality. Additionally, they can learn intricate nonlinear feature representations that capture higher-order and more complex structures and patterns in the data. Before applying hierarchical clustering^23^, we use an autoencoder to reduce the dimensionality of the data and improve the clustering effect. The autoencoder employs a reconstruction loss function, which encourages the model to learn the most informative features of the input data during the encoding process^24^. By minimizing the reconstruction error, the autoencoder effectively selects the most relevant features that capture the underlying patterns in the data. This process serves to further reduce data dimensionality and enhance the overall clustering performance. Subsequently, hierarchical clustering is conducted, treating each subject as an individual cluster. Through the iterative merging of clusters using the Ward connection method, a new cluster hierarchy is gradually formed.

**Figure 2:**
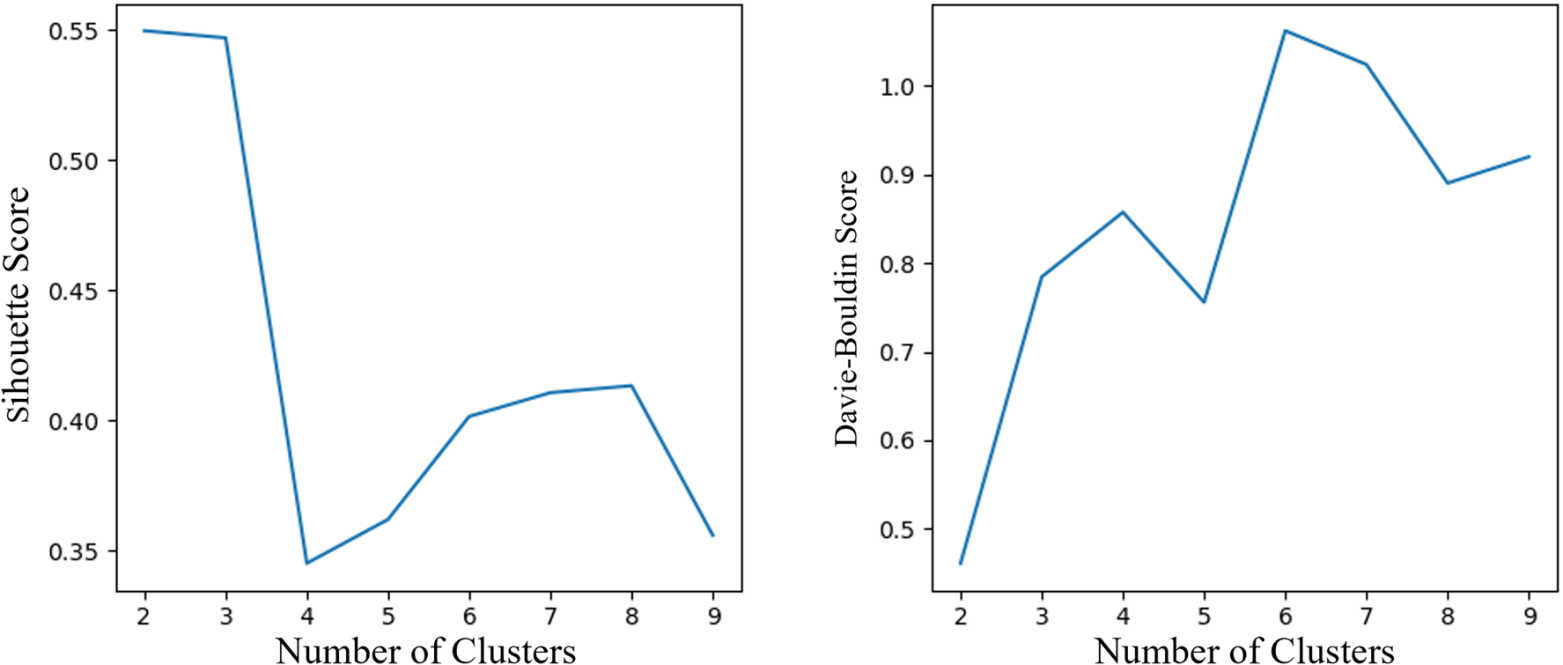
Clustering quality assessment: Silhouette Coefficient and Davies-Bouldin score analysis.

To assess the robustness of the results across different values, we performed the analysis on replicates ranging from 2 to 9. The Silhouette Coefficient^25^ considers both the average distance between data points within clusters and the average distance to data points in neighboring clusters. The Silhouette Coefficient ranges from -1 to 1, with higher values indicating better separation. On the other hand, the Davies-Bouldin Score^26^ evaluates cluster separation by considering the average dissimilarity between each cluster and its most similar neighboring cluster. A lower Davies-Bouldin Score indicates a better clustering performance. The optimal number of clusters ($k=2$), as determined by the joint evaluation of Silhouette Coefficient and Davies-Bouldin Score, is depicted in Fig. 2.

### Intra-network functional connectivity

For each participant, we ranked 360 brain regions based on the Yeo 7 functional networks^27^, and assigned each brain region to the network with which it had the maximum overlap, using the maximum overlap rule^28^. Functional network connectivity for each network was calculated to assess the overall integrity of functional network modules. The intra-network FC^29^ can be expressed by the following Eq. (1),

1 $${Intra-FC}_{X}=\frac{2{\sum }_{i,j\in X,i\ne j}\left|{r}_{ij}\right|}{{n}_{X}({n}_{X}-1)}$$

where ${n}_{X}$ represents the numbers of brain regions contained in the network module, ${r}_{ij}$ represents the correlation coefficient between region $i$ and region $j$. Finally, we examined the correlations between age, education, neuroassessment scale scores, and functional modules.

### Graph theory analysis

False connections or weak connections that connect areas with weaker weights may have less contribution to neural pathways and can introduce noise that affects computational results. Hence, it is necessary to binarize the previously obtained functional connections before computing graph theory parameters. To efficiently identify the optimal threshold for global cost–benefit maximization, the matrix can be subjected to sparse processing using the strongest weight proportional thresholding method. The aim of this sparsification process is to mitigate the influence of spurious connections by filtering out those with weaker weights. The optimal proportion of the strong weights (PSW) aids in determining the optimal threshold that maximizes global cost-effectiveness while excluding connections with lower weights. By enhancing genuine neural pathway connections and reducing false connections caused by noise, we can enhance the reliability and accuracy of data analysis. The mathematical expression for this process is as follows,

2 $$\underset{\mathit{PSW}}{\text{max}}GCE =E-PSW$$

3 $$E=\frac{1}{n}{\sum }_{i\in N}{E}_{i}$$

where $E$ represents the global benefit, ${E}_{i}=\frac{{\sum }_{j\in N,j\ne i}{d}_{ij}^{-1}}{n-1}$ represents the local benefit of the first node in the brain network, $N$ is the set of brain nodes, and ${d}_{ij}$ represents the shortest connected path between node $i$ and node $j$. This paper uses an exhaustive search method to determine the PSW value. We set the search range from 0 to 100%, with a step size of 1%, and record the GCE value for each PSW. Finally, we identify the PSW value corresponding to the maximum GCE value as the threshold used for sparsity processing.

In order to gain a more precise understanding of the functional characteristics of the network and capture the relationship between brain structural properties and functional activities, we employed graph-theoretic metrics on the thresholded matrix. Specifically, we utilized the Brain Connectivity Toolbox^30^ (BCT, available at https://sites.google.com/site/bctnet/) to calculate various indicators, including clustering coefficient, kcoreness, local efficiency and strength. These metrics provide valuable insights into the network's organization and efficiency. Individual differences may increase the variance of the data, thereby masking the true difference signal and making differential analysis more complex and challenging. Individual normalization can eliminate scale and amplitude differences between different individuals, making comparisons between different individuals more comparable. Therefore, we performed individual normalization for each subject. To accurately assess differences in functional connectivity matrices between subtypes, GLM analyses were performed in a univariate manner, incorporating age and sex as covariates. This approach enabled us to examine the specific effects of subtypes while controlling for potential confounding factors. Finally, we employed a false discovery rate (FDR) correction to account for multiple comparisons and control the error rate. Overall, by employing graph-theoretic analysis, GLM modeling, and applying appropriate statistical corrections, we aimed to provide a comprehensive and reliable assessment of the functional connectivity differences between subtypes, accounting for relevant demographic factors and mitigating the risk of spurious associations.

### Ethical approval

This study was approved by the institutional review board (IRB) at Hangzhou Dianzi University (IRB-2020001), and the ethics committee at Beijing Hospital (2022BJYYEC-375–01).

### Consent to participate

Patient consent was waived due to the anonymization of all sensitive information in the collected data.

## Results

### FC-based subtypes of AD

Through multiple repeated experiments, we ensured the stability and reliability of the study, successfully identifying two subtypes. Subsequently, we examined the demographic characteristics of the two subtypes and the control group, which comprised 60 participants (73.17%) in subtype I, 22 participants (26.83%) in subtype II and 50 healthy participants. There are trends in our data to suggest that AD patients were more likely to be classified as subtype I, while they are less likely to be classified as subtype II. The mean age of subtype I was 73.36 years, while subtype II had a mean age of 74.22 years, slightly higher than that for subtype I. Gender distribution was balanced across the three groups. We employed ANOVA analysis, Chi-squared test and Kruskal–Wallis test to examine whether there are differences in age, gender, and scores on the mental scale. The results indicate that there were no significant differences among the three groups in terms of age and gender (both $p>.05$), but there were significant differences in educational level and neuroimaging scores. Table 1 below provides an overview of the participants' demographic and clinical characteristics.

**Table 1:** Demographic and clinical characteristics of AD subtypes.

	Subtype I	Subtype II	CN	*P* value
Age	73.36 (8.24)	74.22 (7.16)	70.74 (6.74)	^a^101
Sex (M/F)	29/31	15/7	21/29	^b^121
Education	15.80 (2.50)	15.41 (2.56)	16.94 (2.02)	^c^012
MMSE	22.55 (3.29)	22.86 (2.85)	29.14 (0.90)	^c^ < 2.2e − 16
RAVLT_immediate	23.18 (7.48)	22.23 (6.29)	49.62 (8.77)	^c^ < 2.2e − 16
RAVLT_learning	1.77 (1.73)	1.95 (1.56)	6.60 (2.43)	^c^ < 2.2e − 16
RAVLT_forgetting	4.75 (1.91)	4.68 (1.81)	3.54 (3.03)	^c^005

Date are presented as either a number or the mean (SD).

^a^One-way ANOVA; ^b^Chi-squared test; ^c^Kruskal-Wallis test.

**Figure 3:**
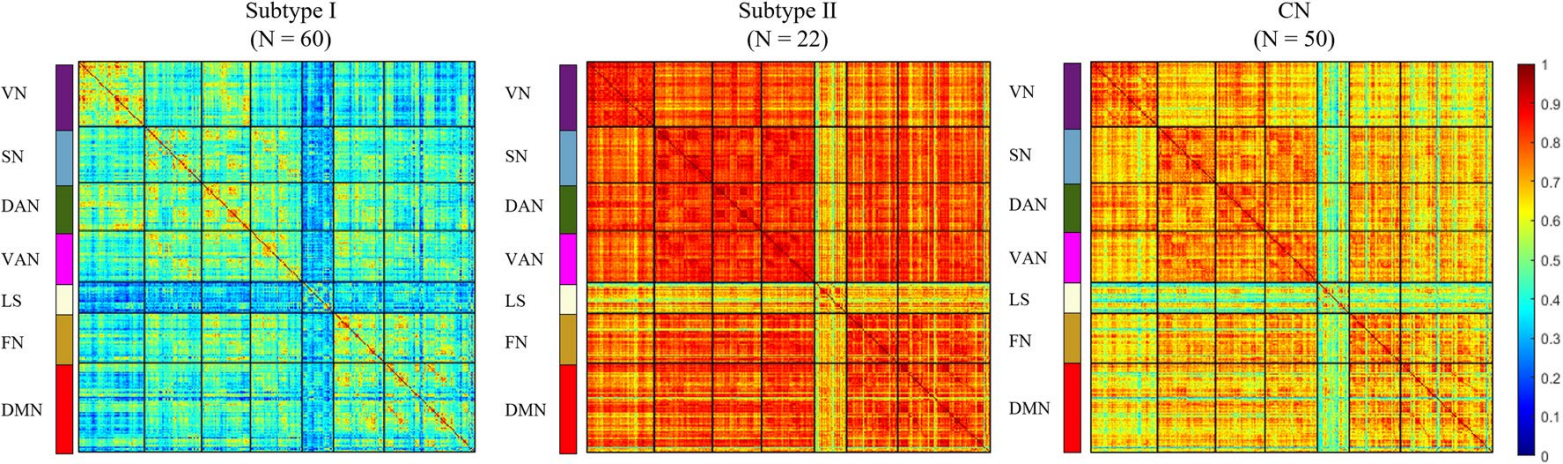
Median FC matrix in each group (N is the number of participants). VN, Visual Network; SN, Somatomotor Network; DAN, Dorsal Attention Network; VAN, Ventral Attention Network; LS, Limbic System; FN, Frontoparietal Network; DMN, Default Mode Network.

Correlation matrices can provide valuable insights into the functional connectivity between different brain regions. Our research revealed a significant heterogeneity in functional connectivity among participants with AD. Despite all of them exhibiting pronounced memory deficits, subtype I display more severe functional disruption across the entire brain, as illustrated in Fig. 3. As age increases, the brain will exhibit varying degrees of atrophy in individuals with CN, accompanied by a certain decline in cognitive abilities. The functional connectivity of subtype II remains largely preserved, displaying similarities with the CN group and even demonstrating stronger connectivity than the CN group. This variation may arise from genetic factors, lifestyle choices, and other cardiovascular and cerebrovascular conditions. Consequently, we have labeled class I patients as the "malignant subtype" and class II patients as the "benign subtype". The median FC matrices exhibited uniform abnormalities in both the LS and its associated resting-state networks (RSN) within the two subtypes. This compelling evidence prompts us to posit that the focal point of the disease may reside within the LS network, highlighting its potential as a critical contributor to the cognitive decline observed in individuals with AD.

**Figure 4:**
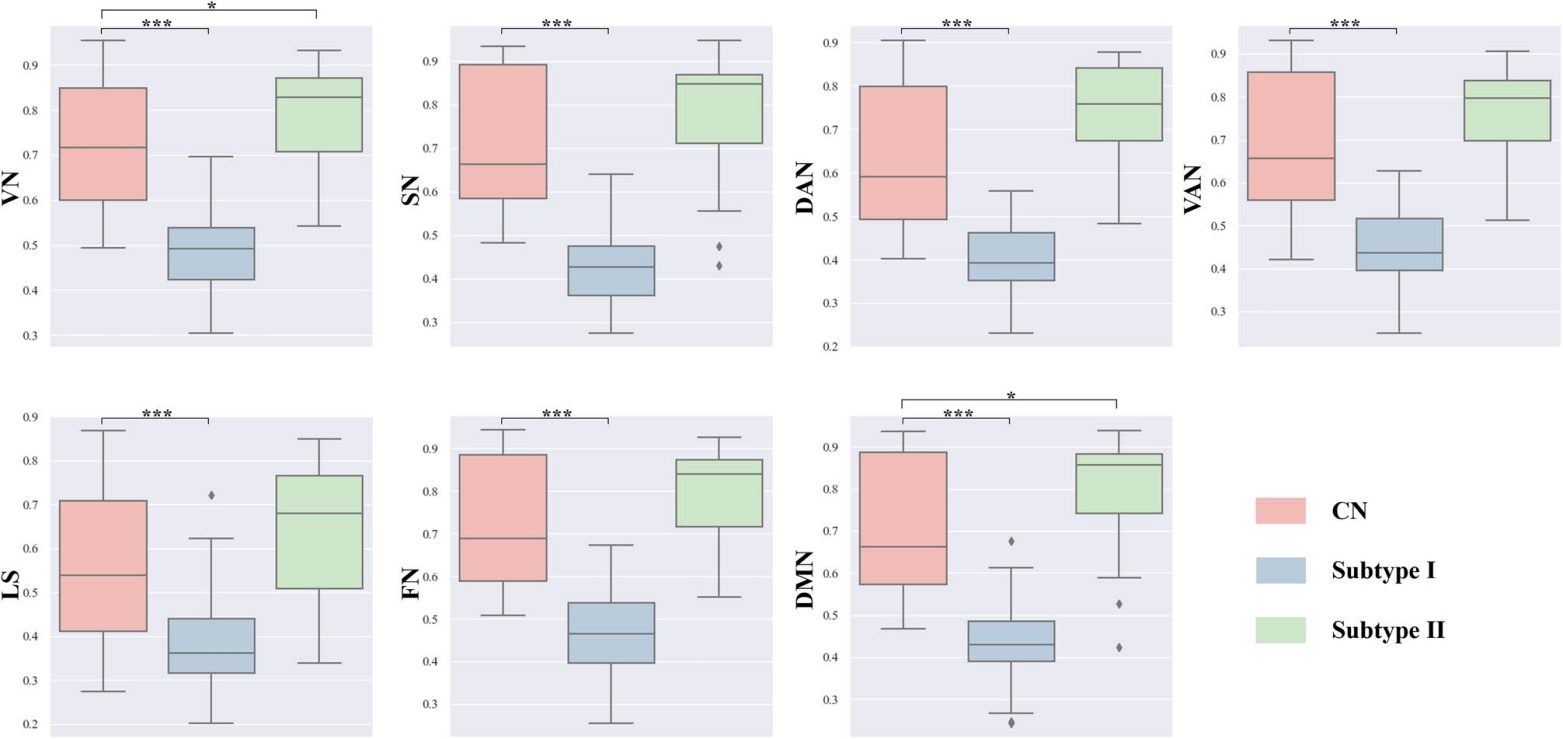
Box plot of intra-network FC.

As shown in Fig. 4, subtype I exhibits significant differences from the control group across all seven brain networks ($p<0.001$), with its median functional connectivity values being lower than both CN and subtype II. This indicates that subtype I has functional connectivity abnormalities across multiple brain networks, which significantly differ from normal aging and are associated with the development of AD.

Compared to this, subtype II showed less pronounced differences in the five functional networks from Sensorimotor Network (SN) to Frontoparietal Network (FN) compared to the control group. However, the differences were relatively significant ($p<0.05$) in the Visual Network (VN) and Default Mode Network (DMN), with subtype II's median functional connectivity higher than CN. The better correlation matrix connectivity in the subtype II group may suggest a higher network density. In the early stages of AD, the brain may compensate for neurodegenerative changes by enhancing the connectivity of certain functional networks. This compensatory mechanism may help maintain certain cognitive functions, even if the brain has undergone atrophy. These findings partially support previous conclusions regarding FC analysis, suggesting that FC in AD may be enhanced^31,32^.

### Association between FC and demographic characteristics

Linear regression is a widely used statistical analysis method. We examined the correlation between patients' demographic characteristics and specific network FC. Within the malignant subtype, we observed a significant negative correlation between Intra-network FC of SN and age ($\text{r}=-0.272,p=0.036$). However, no significant correlation was observed between age and intra-network FC in the benign subtype. Meanwhile, the seven functional connectivities in CN showed a similar significant negative correlation with age. Based on the comprehensive research results, we can conclude that FC within specific brain regions tends to decrease with the increase in age, and this effect varies between the two subtypes.

**Figure 5:**
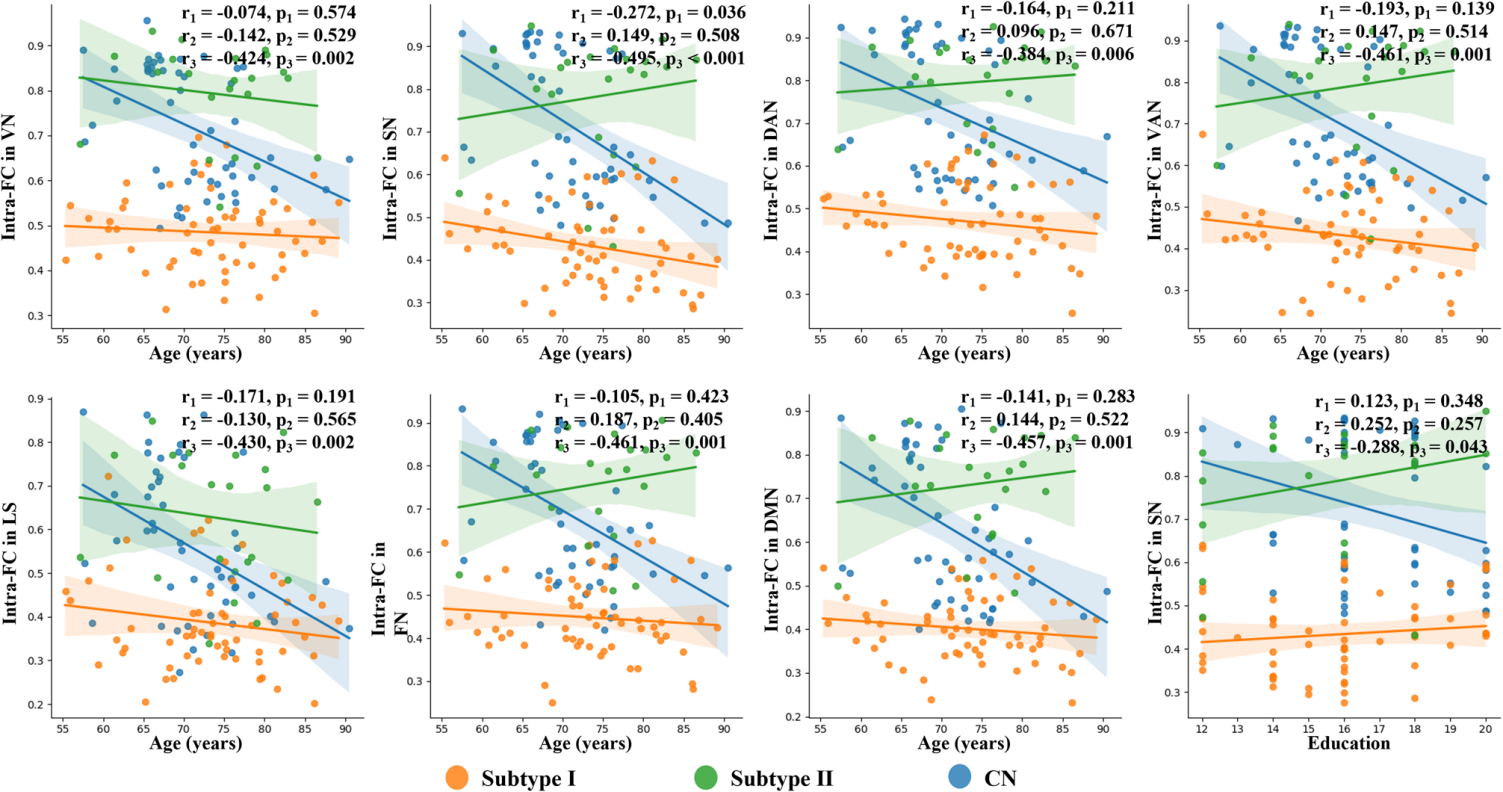
Correlation analysis between intra-network FC values and demographic characteristics. All analyses were conducted using Pearson correlation analysis. The *p* values indicate the level of significance for the observed correlations.

Subsequently, we conducted an analysis to examine the relationship between the intra-network FC and educational level. A positive correlation between the intra-network FC and educational level was observed in the seven functional networks of benign patients. However, it is important to note that this correlation trend did not reach a statistically significant level in the significance test. Further research and a larger sample size may be needed to establish a conclusive relationship between the intra-network FC and educational level in this context. On the other hand, in CN, we discovered a negative correlation ($\text{r}=-0.288, p=0.043$) between the intra-network FC of SN and the educational level. Figure 5 shows the plot of the correlation coefficient between the intra-network FC values and descriptive characteristics.

### Association between FC and cognitive scores

**Figure 6:**
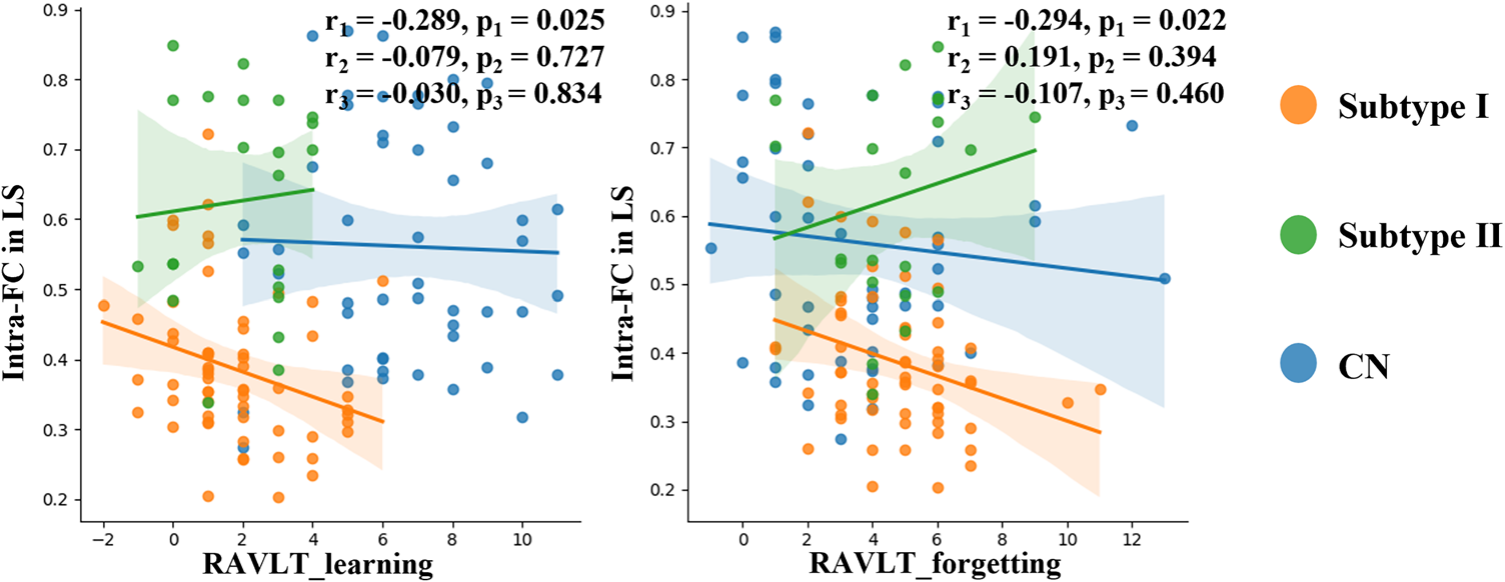
Correlation analysis between intra-network FC values and RAVLT score. All analyses were conducted using Pearson correlation analysis. The *p* values indicate the level of significance for the observed correlations.

MMSE is commonly used to assess an individual's intellectual state and cognitive abilities, while RAVLT is primarily employed to evaluate learning and memory capabilities, particularly in terms of language and speech materials retention. In this section, our investigation centers on exploring the relationship between the scores of MMSE and RAVLT with intra-network FC. As shown in Fig. 6, the malignant subtype of intra-network FC in LS decreased with the RAVLT_learning score increased ($\text{r}=-0.289, p=0.025$), which is not consistent with our perception. Additionally, LS modules were found to be integrated with RAVLT_forgetting scores negatively correlated ($\text{r}=-0.294, p=0.022$). However, no significant correlation was observed between intra-network FC and MMSE and RAVLT series scores in both benign subtypes and CN.

### Graph theory analysis

**Figure 7:**
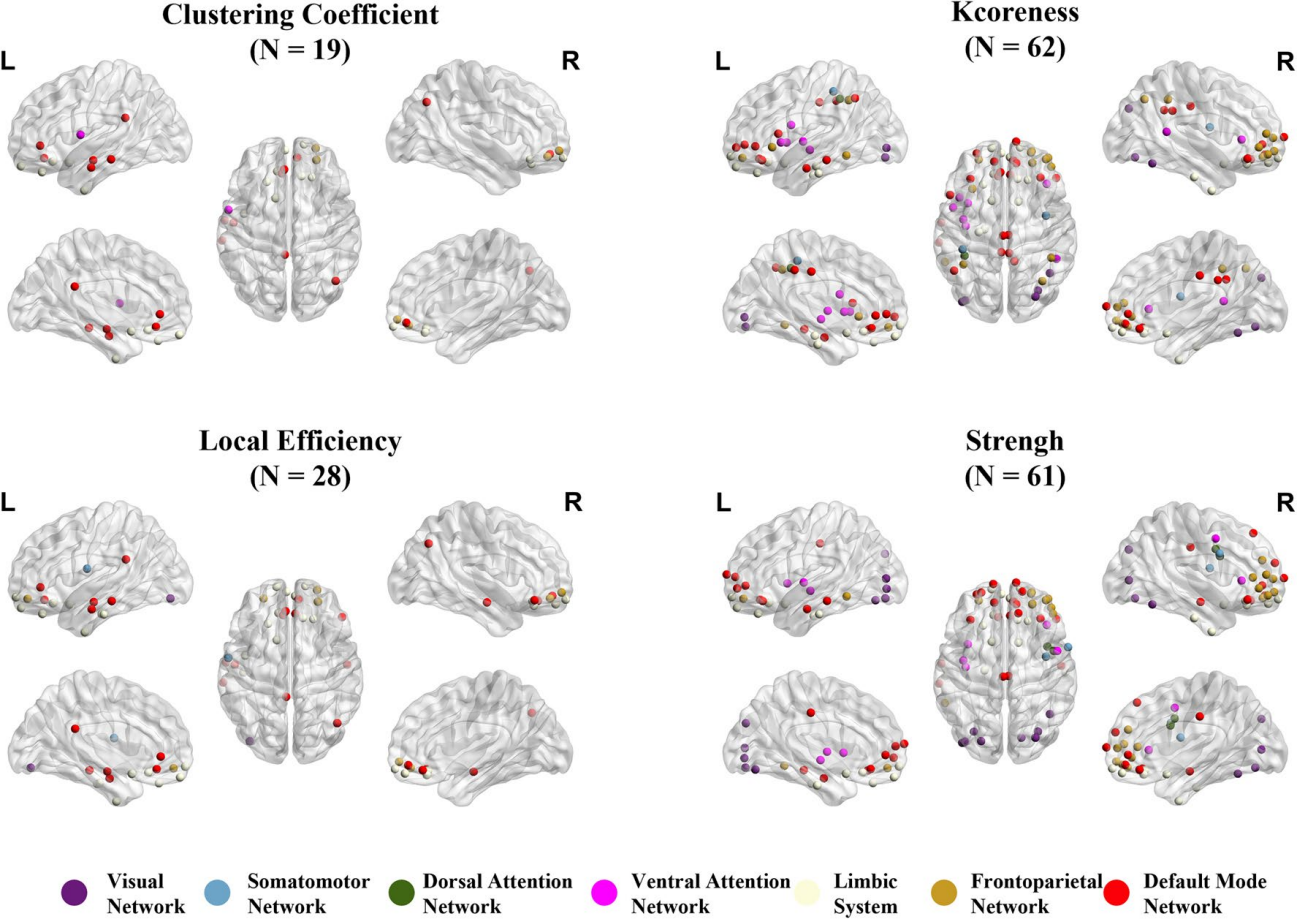
The significant node in the HCPMMP atlas for subtypes is analyzed using graph theory (N is the number of ROIs). Drawing by BrainNet Viewer^36^ is available at https://github.com/mingruixia/BrainNet-Viewer.

**Figure 8:**
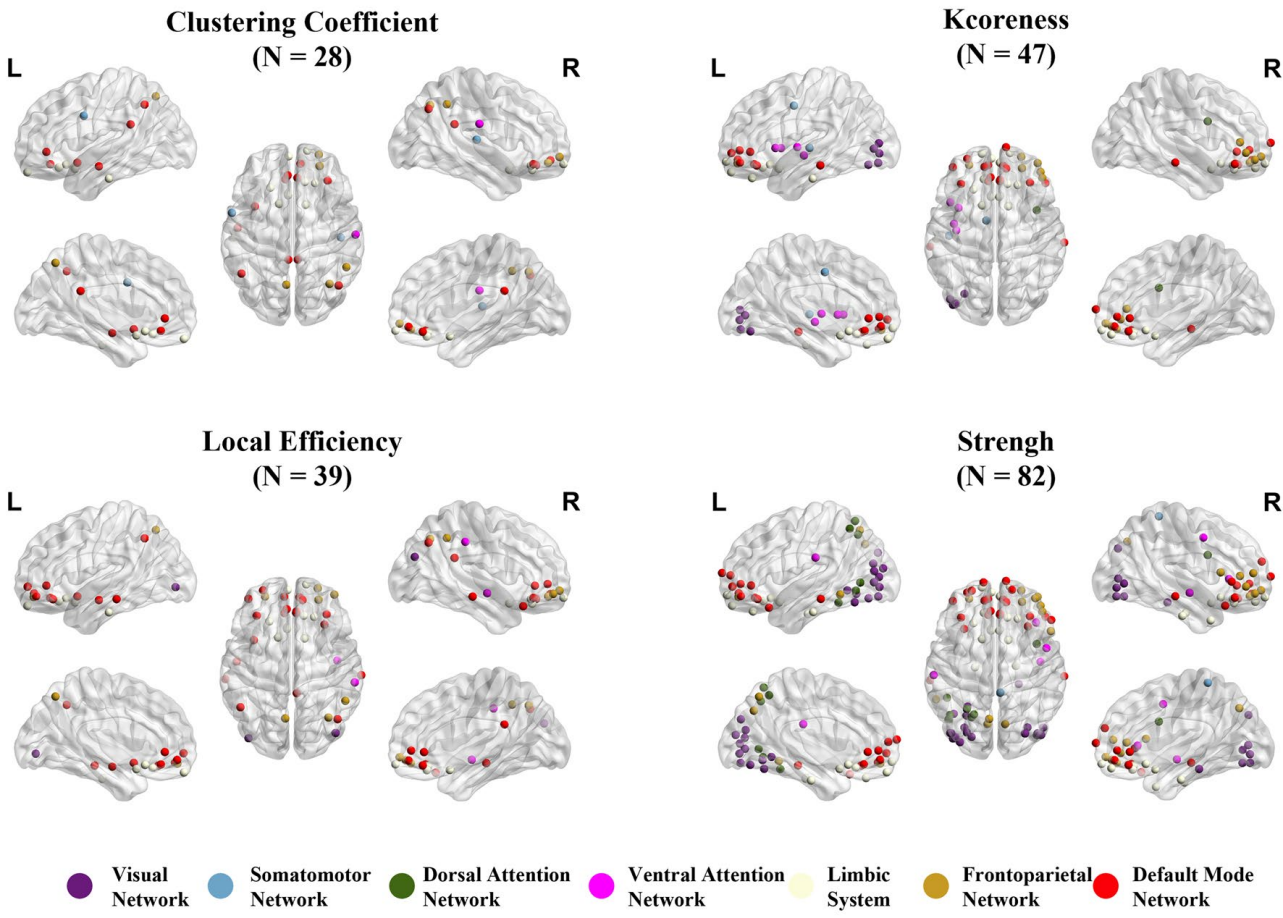
The significant node in the HCPMMP atlas for subtype I and CN is analyzed using graph theory (N is the number of ROIs).

In graph theory, local indicators such as clustering coefficient, kcoreness, and local efficiency are individually analyzed for each brain region, providing insights into the degree of modularity, information transmission capacity, and fault tolerance within relatively independent regions of the brain network^33^. Through GLM analysis of graph theory metrics, we have identified several significant regions in four graph theory metrics, primarily located within the LS and DMN networks, such as OFC^34,35^, and TE1m^12^ brain regions (See Appendix Table A1 for HCPMMP atlas information). In terms of kcoreness and strength metrics, some nodes within FN also exhibit significant differences. Furthermore, many VN nodes are also significant in terms of strength. These significant nodes are illustrated in Fig. 7. Meanwhile, we compared each subtype with the control group. Figure 8 displays the significant brain regions between the control group and subtype I, while subtype II does not have significant nodes compared to CN. Table 2 lists the nodes with significant differences among the four metrics.

**Table 2:** Information on the significant regions in subtypes is defined according to the HCPMMP atlas.

Group	Metrics	Significant regions
Subtype I vs. Subtype II	Clustering coefficient	R_10r, R_a10p, R_10pp, R_11l, R_13l, R_OFC, R_PGs, L_RSC, L_a24, L_10v, L_13l, L_OFC, L_43, L_TE1a, L_s32, L_pOFC, L_TGv, L_STSva, L_TE1m
	Kcoreness	R_V8, R_V7R_FFC, R_23d, R_d23ab, R_31pv, R_p32, R_10r, R_47m, R_10d, R_47l, R_a47r, R_a9-46v, R_10v, R_a10p, R_10pp, R_11l, R_13l, R_OFC, R_FOP2, R_TE2a, R_TPOJ1, R_IP2, R_IP1, R_s32, R_pOFC, R_FOP5, R_p10p, R_p47r, R_TGv, L_LO2, L_PIT, L_23d, L_2, L_a24, L_p32, L_47m, L_44, L_6r, L_10v, L_a10p, L_10pp, L_11l, L_13l, L_OFC, L_PoI2, L_FOP4, L_MI, L_AVI, L_AIP, L_EC, L_H, L_TE1a, L_TE1p, L_TE2a, L_IP2, L_PFm, L_31a, L_s32, L_pOFC, L_PoI1, L_TE1m
	Local efficient	R_10r, R_47l, R_10v, R_a10p, R_10pp, R_11l, R_13l, R_OFC, R_PGs, R_s32, R_STSva, L_RSC, L_PIT, L_3a, L_a24, L_10v, L_10pp, L_11l, L_13l, L_OFC, L_TE1a, L_TE2a, L_TF, L_s32, L_pOFC, L_TGv, L_STSva, L_TE1m
	Strength	R_V1, R_V8, R_PEF, R_55b, R_V7, R_FFC, R_23d, R_6v, R_p32, R_10r, R_47m, R_8BL, R_10d, R_47l, R_a47r, R_IFJp, R_IFSa, R_a9-46v, R_9-46d, R_9a, R_10v, R_a10p, R_10pp, R_11l, R_13l, R_OFC, R_FOP2, R_TE2a, R_s32, R_pOFC, R_FOP5, R_p10p, R_p47r, R_TGv, R_STSva, L_V8, L_V7, L_LO1, L_LO2, L_PIT, L_23d, L_p32, L_47m, L_10d, L_10v, L_a10p, L_10pp, L_11l, L_13l, L_OFC, L_PoI2, L_MI, L_EC, L_TE1a, L_TE1p, L_TE2a, L_s32, L_pOFC, L_PoI1, L_p10p, L_TE1m
Subtype I vs. CN	Clustering coefficient	R_RSC, R_A1, R_10r, R_47m, R_a10p, R_10pp, R_11l, R_13l, R_OFC, R_IP1, R_PFop, R_PFm, R_PGs, R_s32, R_pOFC, L_RSC, L_7Pm, L_6v, L_a24, L_10v, L_13l, L_AAIC, L_TE2a, L_PFm, L_25, L_s32, L_pOFCL_STSva
	Kcoreness	R_a24, R_p32, R_10r, R_47m, R_10d, R_47l, R_a47r, R_IFJp, R_IFSa, R_10v, R_a10p, R_10pp, R_11l, R_13l, R_OFC, R_25, R_s32, R_pOFC, R_p47r, R_TE1m, L_V8, L_LO1, L_LO2, L_PIT, L_24dv, L_a24, L_p32, L_10r, L_47m, L_a47r, L_10v, L_a10p, L_10pp, L_11l, L_13l, L_OFC, L_PoI2, L_FOP4, L_MI, L_TE2a, L_V4t, L_25, L_s32, L_pOFC, L_PoI1, L_MBelt, L_TE1m
	Local efficient	R_RSC, R_a24, R_p32, R_10r, R_47m, R_a47r, R_a10p, R_10pp, R_11l, R_13l, R_OFC, R_47s, R_PGp, R_IP1, R_PF, R_PFm, R_PGs, R_s32, R_pOFC, R_TE1m, R_PI, L_LO2, L_7Pm, L_a24, L_10r, L_47m, L_a47r, L_10v, L_a10p, L_11l, L_13l, L_AAIC, L_TE2a, L_PFm, L_25, L_s32, L_pOFC, L_STSva, L_TE1m
	Strength	R_V8, R_55b, R_POS2, R_V7, R_LO1, R_LO2, R_PIT, R_5m, R_a24, R_p32, R_10r, R_47m, R_10d, R_44, R_45, R_47l, R_a47r, R_IFJp, R_IFSa, R_a9-46v, R_9a, R_10v, R_a10p, R_10pp, R_11l, R_13l, R_OFC, R_47s, R_EC, R_PHA1, R_TGd, R_TE2a, R_V4t, R_25, R_s32, R_pOFC, R_FOP5, R_p47r, R_TE1m, R_PI, L_V8, L_POS2, L_V7, L_FFC, L_V3B, L_LO1, L_LO2, L_PIT, L_7Pm, L_LIPv, L_VIP, L_MIP, L_a24, L_p32, L_10r, L_47m, L_10d, L_a47r, L_10v, L_a10p, L_10pp, L_11l, L_13l, L_OFC, L_47s, L_EC, L_TE1p, L_TE2a, L_TE2p, L_PH, L_PFop, L_VMV3, L_V4t, L_FST, L_V3CD, L_LO3, L_VMV2, L_25, L_s32, L_pOFC, L_p10p, L_TE1m

## Discussion

Existing heterogeneity studies have predominantly relied on sMRI or PET imaging^37^. This study, utilizing fMRI imaging, has successfully identified two subtypes of AD, namely, the "malignant subtype" and the "benign subtype". These two AD subtypes exhibit significant differences in FC, suggesting that AD is not a singular disease entity, and specific subtypes may be more responsive to certain drugs or treatment approaches.

Firstly, we noticed that the malignant subtype exhibits extensive functional connectivity loss, while the benign subtype only shows mild impairments within the LS and its associated RSNs. We hypothesize that the compensatory mechanisms may account for the better performance of FC in the benign subtype, while the LS may be the primary affected area in the disease. The first area of the brain to be impacted in early AD (benign subtype) is the DMN, which is also the main reason for memory loss. This damage gradually affects additional networks as the illness worsens, turning it into a malignant subtype. LS, which includes key structures such as the hippocampus, cingulate gyrus, and amygdala, plays a crucial role in regulating the generation, expression, and control of emotions, as well as in the formation and storage of memories^38,39^. Early-stage AD is typically accompanied by neuronal loss and inflammatory responses. Detecting changes in LS (biomarker) through biomarker testing can serve as an early indicator of AD, aiding in early diagnosis and intervention. Previous studies have indicated that the disruption of structural network topology in MCI and AD patients primarily occurs in regions within LS^40^. Enhancing connectivity within LS may potentially help maintain memory function, possibly serving as a compensatory mechanism^41^. This aligns with our findings.

Secondly, the findings show a significant negative correlation between intra-network FC of SN and age in the malignant subtype. These findings suggest that in both malignant and benign subtypes, the internal connectivity of brain networks may be influenced by age, and this influence may differ between the two subtypes. Subtype II exhibits higher FC and positive correlation after a specific age, although not significantly, but it still reflects the potential initiation of certain compensatory mechanisms. The role of age in functional connectivity and correlation is a complex and important variable. During aging, these compensatory mechanisms may enhance functional connectivity to maintain cognitive function or other brain activities. This finding contributes to providing potential clues for personalized therapy. For instance, treatment for subtype I patients may require more attention to the stability of neural networks, whereas therapy for subtype II patients may involve strategies to support and enhance brain compensatory mechanisms.

Third, RAVLT_forgetting score measures the short-term memory capacity of the subjects, with lower scores indicating that the subjects are better at retaining and recalling learned information in delayed recall tasks. We have identified a negative correlation between LS modules and RAVLT_forgetting scores. In addition, we have also uncovered some intriguing findings. The intra-network FC of the malignant subtype LS decreases as the RAVLT_learning score increases, which is inconsistent with our initial expectations^42^. We speculate that patients with the malignant subtype of AD may exhibit poorer adaptability in learning and memory, requiring more cognitive effort to remember these words. In other words, when the FC value has already fallen below a certain threshold, additional cognitive burden may render their brains unable to maintain normal functional connections, resulting in a reduction in intra-network FC in the default mode network. Of course, this is just an intriguing hypothesis, and the actual reasons may be more complex, warranting further research for a deeper investigation.

Finally, there are significant differences in graph-theoretical metrics. DMN is closely associated with memory retrieval and mind-wandering, primarily involving brain regions such as the frontal lobe, temporal lobe, and posterior cingulate cortex^43^. Compared to the benign subtype/CN, the malignant subtype shows significant differences in clustering coefficient, k-coreness, local efficiency, and node strength in many nodes within DMN and LS. These regions are involved in normal information transmission and processing functions. Additionally, significant nodes are also present in FN and VN. FN and VN are responsible for advanced cognitive and visual information processing. Impairment of their functions leads to difficulties in decision-making and visual processing in patients. In summary, the significant differences in these graph theory metrics reflect pronounced distinctions in brain network structure and function between malignant subtypes and benign subtypes.

This study has several limitations that need to be addressed. Firstly, the sample size is relatively small, and in future studies, it should be expanded to increase the reliability of our conclusions. Secondly, while we have identified differences in functional connectivity between the two subtypes, the relationship between these differences and other factors, such as gene variations and drug effects, remains unclear. Therefore, further in-depth research is necessary to explore the impact of these factors on the differences between subtypes. Future research should incorporate multimodal brain imaging data, genomics and long-term follow-up data to delve into the subtype differences of AD and their relationship with clinical manifestations. This will enable us to gain a more comprehensive understanding of the essence of AD and discover the key factors affecting AD cognition in the AD process.

## Conclusion

In this article, we have identified significant heterogeneity among subjects in the degree of disruption in two sets of functional connections: a benign subtype and a malignant subtype. We hypothesize that the benign subtype represents a pre-malignant state, characterized by mild impairments only observable in LS and its associated RSNs. The biological criteria of LS may serve as an early indicator for AD. Furthermore, we have observed a novel negative correlation between intra-network FC in the malignant subtype of LS and RAVLT_learning. We speculate that as FC values decline to a certain extent, the burden of memory and learning leads to a reduction in the functional connectivity of LS. This study not only sheds light on the differences in functional connectivity between AD subtypes but also offers novel perspectives for predicting, diagnosing, and treating AD. Further research on the distinctions between these subtypes, as well as the molecular and physiological mechanisms underlying the decline of LS function, will deepen our understanding of the complex nature of AD and provide robust support for developing more effective treatment strategies. It is essential to acknowledge that additional research is necessary to verify and refine these findings, which may pave the way for innovative approaches in the treatment and management of AD.

## Data Availability

The data supporting this research is derived from the Alzheimer's Disease Neuroimaging Initiative (ADNI) and can be accessed from the ADNI database (adni.loni.usc.edu) upon registration and adherence to the data usage agreement. Additional information can be provided by the corresponding upon reasonable request.

## Supplementary Table

Supplementary Tables.
