# Supplementary material for: Novel Alzheimer's disease subtypes based on functional brain connectivity in human connectome project
Source: Sci Rep. 2024 Jun 27;14:14821. doi: 10.1038/s41598-024-65846-z (PMC11211325; doi:10.1038/s41598-024-65846-z)
Supplement: Supplementary file 1 — Supplementary Tables. [file 41598_2024_65846_MOESM1_ESM.pdf]

Supplementary material

Table A1. Summary of the HCPMMP Atlas.

Name	Area Description	Name	Area Description
V1	Primary Visual Cortex	11l	Area 11l
MST	Medial Superior Temporal Area	13l	Area 13l
V6	Sixth Visual Area	OFC	Orbital Frontal Complex
V2	Second Visual Area	47s	Area 47s
V3	Third Visual Area	LIPd	Area Lateral IntraParietal Area
V4	Fourth Visual Area	6a	Area 6 anterior
V8	Eighth Visual Area	i6-8	Inferior 6-8 Transitional Area
4	Primary Motor Cortex	s6-8	Superior 6-8 Transitional Area
3b	Primary Motor Cortex	43	Area 43
FEF	Frontal Eye Fields	OP4	Area OP4/PV
PEF	Premotor Eye Fields	OP1	Area OP1/SII
55b	Area 55b	OP2-3	Area OP2-3/VS
V3A	Area V3A	52	Area 52
RSC	RetroSplenial Complex	RI	RetroInsular Cortex
POS2	Parieto-Occipital Sulcus Area 2	PFcm	Area PFcm
V7	Seventh Visual Area	Pol2	Posterior Insular Area 2
IPS1	IntraParietal Sulcus Area 1	TA2	Area TA2
FFC	Fusiform Face Complex	FOP4	Frontal OPercular Area 4
V3B	Area V3B	MI	Middle Insular Area
LO1	Area Lateral Occipital 1	Pir	Pirform Cortex
LO2	Area Lateral Occipital 2	AVI	Anterior Ventral Insular Area
PIT	Posterior InferTemporal	AAIC	Anterior Agranular Insular Complex
MT	Middle Temporal Area	FOP1	Frontal OPercular Area 1
A1	Primary Auditory	FOP3	Frontal OPercular Area 3
PSL	PeriSylvian Language Area	FOP2	Frontal OPercular Area 2
SFL	Superior Frontal Language Area	PFt	Area PFt
PCV	PreCuneus Visual Area	AIP	Anterior IntraParietal Area
STV	Superior Temporal Visual Area	EC	Entorhinal Cortex
7Pm	Medial Area 7P	PreS	PreSubiculum
7m	Area 7m	H	Hippocampus
POS1	Parieto-Occipital Sulcus Area 1	ProS	ProStriate Area
23d	Area 23d	PeEc	Perirhinal Ectorhinal Cortex
v23ab	Area ventral 23 a+b	STGa	Area STGa
D23ab	Area dorsal23 s+b	PBelt	ParaBelt Complex
31pv	Area 31p ventral	A5	Auditory 5 Complex
5m	Area 5m	PHA1	ParaHippocampal Area 1
5mv	Area 5m ventral	PHA3	ParaHippocampal Area 3

23c	Area 23c	STSda	Area STSs anterior
5L	Area 5L	STSdp	Area STSd posterior
24dd	Dorsal Area 24d	STSvp	Area STSv posterior
24dv	Ventral Area 24d	TGd	Area TG dorsal
7AL	Lateral Area 7A	TE1a	Area TE1 anterior
SCEF	Supplementary and Cingulate Eye Field	TE1p	Area TE1 posterior
6ma	Area 6m anterior	TE2a	Area TE2 anterior
7Am	Medial Area 7A	TF	Area TF
7PI	Lateral Area 7P	TE2p	Area TE2 posterior
7PC	Area 7PC	PHT	Area PHT
LIPv	Area Lateral IntraParietal ventral	PH	Area PH
VIP	Ventral IntraParietal Complex	TPOJ1	Area TemporoParietoOccipital Junction 1
MIP	Medial IntraParietal Area	TPOJ2	Area TemporoParietoOccipital Junction 2
1	Area 1	TPOJ3	Area TemporoParietoOccipital Junction 3
2	Area 2	DVT	Dorsal Transitional Visual Area
3a	Area 3a	PGp	Area PGp
6d	Dorsal area 6	IP2	Area IntraParietal 2
6mp	Area 6mp	IP1	Area IntraParietal 1
6v	Ventral Area 6	IP0	Area IntraParietal 0
p24pr	Area Posterior 24 prime	PFop	Area PF opercular
33pr	Area 33 prime	PF	Area PF Complex
a24pr	Anterior 24 prime	PFm	Area PFm Complex
p32pr	Area p32 prime	PGi	Area PGi
a24	Area a24	PGs	Area PGs
d32	Area dorsal 32	V6A	Area V6A
8BM	Area 8BM	VMV1	VentroMedial Visual Area 1
p32	Area p32	VMV3	VentroMedial Visual Area 3
10r	Area 10r	PHA2	ParaHippocampal Area 2
47m	Area 47m	V4t	Area V4t
8Av	Area 8Av	FST	Area FST
8Ad	Area 9Ad	V3CD	Area V3CD
9m	Area 9 Middle	LO3	Area Lateral Occipital 3
8BL	Area 8B Lateral	VMV2	VentroMedial Visual Area 2
9p	Area 9 Posterior	31pd	Area 31pd
10d	Area 10d	31a	Area 31a
8C	Area 8C	VVC	Ventral Visual Complex
44	Area 44	25	Area 25
45	Area 45	s32	Area s32
47l	Area 47l (47 lateral)	pOFC	Posterior OFC Complex
a47r	Area anterior 47r	PoI1	Area Posterior Insular 1
6r	Rostral Area 6r	Ig	Insular Granular Complex
IFJa	Area IFJa	FOP5	Area Frontal Opercular 5
IFJp	Area IFJp	p10p	Area posterior 10p

IFSp	Area IFSp	P47r	Area posterior 47r
IFSa	Area IFSa	TGv	Area TG Ventral
p9-46v	Area posterior 9-46v	MBelt	Medial Belt Complex
46	Area 46	LBelt	Lateral Belt Complex
a9-46v	Area anterior 9-46v	A4	Auditory 4 Complex
9-46d	Area 9-46d	STSva	Area STSv anterior
9a	Area 9 anterior	TE1m	Area TE1 Middle
10v	Area 10v	PI	Para-Insular Area
a10p	Area anterior 10p	a32pr	Area anterior 32 prime
10pp	Polar 10p	P24	Area posterior 24
